# A Double Siamese Framework for Differential Morphing Attack Detection

**DOI:** 10.3390/s21103466

**Published:** 2021-05-16

**Authors:** Guido Borghi, Emanuele Pancisi, Matteo Ferrara, Davide Maltoni

**Affiliations:** DISI—Dipartimento di Informatica-Scienza e Ingegneria, Università di Bologna, 47521 Cesena, Italy; emanuele.pancisi@studio.unibo.it (E.P.); matteo.ferrara@unibo.it (M.F.); davide.maltoni@unibo.it (D.M.)

**Keywords:** face morphing, morphing attack detection, differential morph attack detection, single-image morph attack detection, deep learning, Siamese networks

## Abstract

Face morphing and related morphing attacks have emerged as a serious security threat for automatic face recognition systems and a challenging research field. Therefore, the availability of effective and reliable morphing attack detectors is strongly needed. In this paper, we proposed a framework based on a double Siamese architecture to tackle the morphing attack detection task in the differential scenario, in which two images, a trusted live acquired image and a probe image (morphed or bona fide) are given as the input for the system. In particular, the presented framework aimed to merge the information computed by two different modules to predict the final score. The first one was designed to extract information about the identity of the input faces, while the second module was focused on the detection of artifacts related to the morphing process. Experimental results were obtained through several and rigorous cross-dataset tests, exploiting three well-known datasets, namely PMDB, MorphDB, and AMSL, containing automatic and manually refined facial morphed images, showing that the proposed framework was able to achieve satisfying results.

## 1. Introduction

Recently, morphing attacks have raised the interest of researchers belonging to different research areas, ranging from biometrics to computer vision [1,2]. Indeed, it has been proven [3] that morphing attacks represent a concrete and serious threat for various applications relying on identity verification: for instance, this is the case of the *Automated Border Control* (ABC) gates often present in international airports, which automatically verify if the face photo stored in the *electronic Machine Readable Travel Document* (eMRTD), such as an e-passport, corresponds to the face of the document owner. Through a face morphing operation, which consists of merging two identities into a single face, two subjects can share the same legal document, e.g., the passport or the ID card. In particular, a subject with no criminal records (here referred to as *accomplice*) could apply for an official document presenting a photo morphed with the face of a *criminal*. Then, the criminal can use that document to elude identity-based controls. Here, it is important to note that the resulting document is perfectly regular, and it can be used to fool both the human control (the police officer) and the current *Commercial-Off-The-Shelf* (COTS) *Face Recognition Systems* (FRSs). Moreover, thanks to the wide spread of ready-to-use face morphing tools, such as *FaceFusion* [4], *FaceMorpher* [5], and *UBO-Morphed* [6] also a criminal without specific knowledge can easily obtain high-quality morphed images: in this scenario, the main obstacle is finding a similar subject, in terms of visual appearance.

Therefore, in this context, methods that tackle the task of *Morphing Attack Detection* (MAD), i.e., algorithms that are able to automatically and accurately detect morphed face images, are strongly demanded [7]. These systems are expected to cooperate with existing FRSs, providing a score that reveals if an input image is *bona fide* or not (*morphed*).

**Figure 1:**
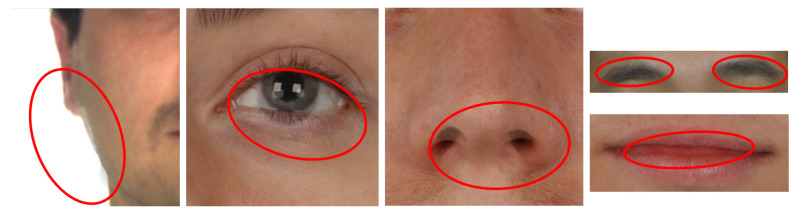
Examples of artifacts usually produced by morphing algorithms. These artifacts are produced by an inaccurate detection of the facial landmarks that are used during the morphing procedure. Ghosts, blurred areas, and texture inconsistency are visible and highlighted with red circles.

In this paper, we proposed an MAD framework based on a double Siamese architecture. The underlying idea is to develop a framework consisting of two deep learning-based modules, the former (here referred to as the “Identity” block) specialized in the extraction of identity information and the latter (“Artifact” block) designed to detect the presence of artifacts produced by the morphing process. A final element aimed to merge these two sources of information to output the final prediction. The framework receives as the input a couple of facial images and predicts a score that indicates the presence of a morphed face image or not. Each module works on both input images. Specifically, the first module compares the two input faces and computes a face verification score, while the second one detects artifacts located, for instance, in the proximity of the main facial features, such as the nose, the mouth, and the eyes (some examples are shown in Figure 1). These artifacts are usually produced by a misalignment of the facial landmarks during the morphing procedure.

## 2. Related Work

In the field of morphing attack detection systems, two main scenarios can be distinguished relying on the number of input images: *Single-image* (S-MAD) and *Differential* (D-MAD) methods [8]. Hereafter, we briefly analyzed these two types of approaches, focusing more on differential methods to which the proposed method belongs.

### 2.1. Differential Morphing Detection Attack

Differential methods, also referred to as *two image-* or *pair*-based methods, receive a couple of face images as the input. The task consists of deciding if the first image (*probe*) is morphed or not; the second image (which is a *trusted live capture*) can be used as a task helper (e.g., compared with the first one to point out inconsistencies). These two images can be acquired during, for instance, the passport issuance in which the first image is the photo provided and the second is the live acquisition; also, during the controls at ABCs, the live image is acquired through the automated face verification procedure, and the probe is the image stored in the eMRTD.

A first simple approach to tackle this task was described in [9]: a comparison between the facial landmarks extracted in bona fide and morphed images is carried out. Differences are expressed in terms of the Euclidean and angle distance, and the best results, although not very satisfactory, are obtained using a *Support Vector Machine* (SVM) [10] with a *Radial Basis Function* (RBF) kernel. The method proposed in [11] aimed to invert the morphing process to detect if more than one identities are merged in the same face. Experimental results revealed an overall good accuracy, even though the main issue was represented by the fact that usually the morphing process is not a simple linear combination. Following a similar idea, in [12], a face de-morphing *Generative Adversarial Network* (GAN)-based method was proposed to restore the accomplice’s facial image, starting from a couple of images. The GAN framework, referred to as FD-GAN, is based on symmetric dual-network architectures and two levels of restoration losses. The state-of-the-art in the MAD scenario is currently represented by the recent work of Scherag et al. [13]: a CNN network trained to solve the face recognition task, i.e., a ResNet architecture trained with an angular margin loss [14], here merely referred to as *ArcFace*, is used to extract visual features from both input images. As stated by the authors, the network was pre-trained on the original datasets, and no further training procedures were conducted on the network; this element ensures the lack of overfitting of the deep learning model to the morphing detection task. The extracted features were subtracted and passed to an SVM for the final classification. We note that this approach tackled the D-MAD scenario exploiting only the differences in terms of identity between the accomplice and the criminal; potentially visible artifacts related to a morphing process applied on the input images were not directly used to predict and consolidate the final score. Recently, a variety of anthropometry-based features were proposed in [15], trying to capture the craniofacial proportions, and used as the input for an SVM classifier. In [16], a deep learning-based architecture was trained by triplets of face images, employing the landmark disentanglement and subjects’ appearance. Specifically, this approach computes the final score using the distances between landmarks, the appearance (consisting of two different, but complementary embedded representations), and the ID information.

### 2.2. Single-Image Morphing Detection Attack

Single-image methods, also referred to as *no-reference* or *forensic* methods, receive only one face image as the input. Thus, the morphing attack detection is conducted on a single image that can be the photo presented to the police officer during the enrollment procedure or the face read from the eMRTD during the controls, for instance at the ABC gates. These approaches work under the assumption that the morphing procedure leaves specific anomalous patterns and traces on the processed images, in terms of artifacts, texture anomalies, and other visual clues. A large majority of literature works belong to this approach, which is usually considered more challenging with respect to the differential scenario. A seminal work [17] aimed to extract discriminative texture features through traditional approaches, i.e., *Local Binary Patterns* (LBPs) [18], *Binarized Statistical Image Features* (BSIFs) [19], and the *Histogram of Oriented Gradient* (HOG) [20], classifying them through an SVM with an RBF kernel. However, the final results were limited in accuracy. Similarly, authors of the work [21] proposed the combined use of HOG, LBP, and *Scale-Invariant Feature Transform* (SIFT) [22] features extracted from grayscale 320×320 images. Different SVMs were then trained for each descriptor, and the final scores were merged through a score-level fusion strategy. Experimental results showed that the combined use of different descriptors improved the performance of single descriptors, but a manual fine-tuning of each descriptor’s hyper-parameters was strongly required. A feature fusion method to detect morphed images was proposed in [23], in which features were extracted through two different deep learning networks, i.e., *VGG19* [24] and *AlexNet* [25]. The authors stated that two different neural networks, even though trained on the same dataset, could provide complementary features that were finally classified by a *Probabilistic Collaborative Representation Classifier* (P-CRC). Other works proposed to use different types of descriptors (instead of the aforementioned hand-crafted features) and approaches. Zhank et al. [26] proposed to compute the Fourier spectrum of sensor pattern noise to detect morphing in images. On an input pre-processed image, a quantized statistics feature was extracted and then classified as normal or morphed by an SVM. Similarly, Scherag et al. [27] proposed to extract spatial and spectral features from the *Photo Response Non-Uniformity* (PRNU). After a feature aggregation phase, a final decision was made by a simple threshold-based operation. In [28], the authors analyzed the correctness of the illumination, estimating the direction of the light sources. These estimations were then compared with the synthetic reference: bad alignments and different geometries indicated the presence of morphing.

### 2.3. General Considerations

One of the main obstacles in the development of MAD algorithms is the lack of publicly released datasets, with a great data variety in terms of the number of subjects and morphing tools exploited. This is mainly due to privacy constraints. This leads to some criticalities, as reported in [2]. Indeed, there is a need for cross-dataset evaluations. All the above-reported S-MAD and D-MAD works presented a good level of performance in in-house experiments, but they were usually evaluated on limited sets, in which the testing data were a sub-sample of the dataset used for the training and validation phases. Only a few works [7,29] tried to investigate the generalization capabilities of their proposed approach. Therefore, in this paper, we evaluated our method with a cross-dataset modality, training and validating the proposed approach on a dataset, and testing the framework on two different ones. In addition, we avoided tuning our method on particular a priori considerations related to the type of the morphing tool used and the quality of the input images.

## 3. Proposed Method

**Figure 2:**
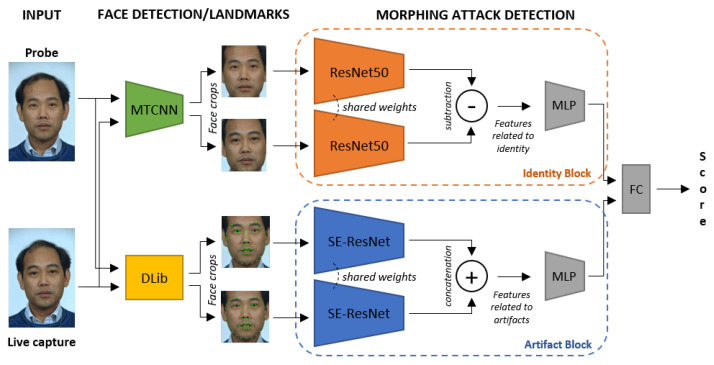
Overview of the proposed framework working in the D-MAD scenario. As shown, the framework was mainly based on two modules: the first module, referred to as the “Identity” block, focuses on the subjects’ identity of the input images, computing a sort of face verification score; the second one, namely the “Artifact” block, is based on the analysis of artifacts produced by morphing operations, such as ghosts, blurred areas, and texture inconsistencies (as shown in Figure 1). The features computed through these modules are then merged by the final fully connected layer to output the score that reveals whether an image is morphed or not.

In this section, we report the details of the proposed method—which is based on a double Siamese architecture—the pre-processing of input images, and the adopted training procedure. An overview of the proposed framework is depicted in Figure 2. As shown, working in the D-MAD scenario, it receives as the input a couple of images. The proposed framework consisted of two different modules, referred to as “Identity” (orange) and “Artifact” (blue) blocks, respectively, and each block was based on a Siamese network followed by a MultiLayer Perceptron (MLP) that act as fusion layers. Finally, a Fully Connected layer (FC) merges the features originated from the two modules and outputs the final score.

### 3.1. Data Pre-Processing

The framework is fed with a pair of RGB facial images, a trusted live capture and a probe image. Then, the first step is to detect the face in order to isolate the face area from the background. This operation is based on two different face detection methods. Following the original implementation described in [14], the Identity block relies on the MTCNN detector [30], based on a multi-task cascade structure with three deep convolutional neural networks that predict face and landmark locations. The input size of the MTCNN network and of this module is 112 × 112 pixels. Instead, for the Artifact block, the method contained in DLib libraries [31] is exploited; it is based on the computation of HOG features [20] and the adoption of a linear classifier. A sliding window detection scheme is employed in combination with an image pyramid system in order to improve the scale invariance. The DLib detector is able to predict up to 68 landmarks, organized following the MPEG-4 standard, a higher number compared to the 5 landmarks provided by the MTCNN detector adopted. After the computation of facial landmarks, we cropped the face area adding a 20% offset in width and height with respect to the initial facial landmark position. Moreover, the use of the DLib detector permitted obtaining more accurate facial landmark locations on the face, especially for the jawline part, needed to correctly crop the frontal face, and to directly work on images with full spatial resolution. Finally, input face images of the Artifact block were resized to a spatial resolution of 224 × 224 pixels.

### 3.2. Double Siamese Architectures

As mentioned before, from a general point of view, the framework is divided into two blocks, as shown in the right part of Figure 2. Each block is based on a Siamese network, i.e., a neural network with two branches that share the same architecture and weights and that are finally merged in a single deep architecture (in our case, through an MLP). The two blocks were designed to focus on different types of features. The Identity block, inspired by the works proposed in [13,14], was based on a CNN trained on huge RGB datasets for the face recognition task. Then, we expected that this block would be able to extract highly discriminative features that were related to the identity of the input subjects. The second element, referred to as the Artifact block, was instead based on a CNN specifically trained to extract features that were directly related to the presence of artifacts that could be produced by a generic face morphing algorithm.

#### 3.2.1. Identity Block

In this block, we adopted a state-of-the-art *ResNet50* [32] architecture as the backbone, trained with the *additive angular margin loss* [14] for the face recognition task, on MS-Celeb-1M [33] and VGGFace2 [34], large-scale datasets with trillions of face pairs. Its performance consistently outperformed current face recognition approaches available in the literature: the two branches of the Siamese network were based on this architecture and shared their weights. From an implementation point of view, the last fully connected layer was removed, and the feature maps from the last convolutional layer were extracted. Indeed, given two input facial images, the two branches output two feature maps with a size of 512, which are then combined through a subtraction operation, preserving the dimension. The resulting feature map is fed into a multi-layer perceptron, composed of three fully connected layers with 1024, 512, and 2 neurons, respectively. Dropout regularization (p=0.5) was applied on the first two layers of the MLP.

#### 3.2.2. Artifact Block

This block was based on the recent *SE-Resnet50* [35] architecture, originally proposed for the image classification task. Similar to the previous block, the two branches of the Siamese network were both based on this architecture and shared their weights. Given two input facial images, two different feature maps of size 2048 were computed. Different from the previous case, these features were then concatenated, achieving a final dimension of 4096. Indeed, in the Identity block, the identity features, whether computed on input images that belong to the same subject, are “symmetrical”, and then the result of the subtraction procedure can reveal the presence of a second identity. On the contrary, the features related to the presence of artifacts are not “symmetrical”, since morphing artifacts are present only on one image, and then, in this case, the concatenation operation is more discriminative for the final classifier. Moreover, features extracted from the trusted live image (in which morphing was not applied) represent a touchstone, useful to filter out artifacts related, for instance, to the quality of the image. Internal experimental results confirmed the effectiveness of this approach. The last part of the Siamese model is represented by an MLP architecture with three fully connected layers of size 1024, 512, and 2, respectively. Dropout regularization was applied on the first two layers with p=0.5.

### 3.3. Training Procedure

The outputs produced by the two blocks were finally concatenated and used as the input for the last fully connected layer with 2 neurons. Then, the final output was represented by a score in the range [0, 1], i.e., the probability if the probe input image was bona fide or morphed. From a general point of view, there were five networks that composed the framework and that were to be trained: for each Siamese block, the backbone architectures (2 networks since the two branches shared the weights), the two MLPs at the end of blocks (2 networks), and the final fully connected layer. The training of the whole framework followed a two-step procedure.

In the first step, each architecture used as a backbone in the Siamese networks was individually trained. In the case of ResNet50, we followed the authors’ implementation [36] in order to guarantee the full reproducibility of the performance obtained in the original paper, exploiting the additive angular margin loss [14]. We used *MXNet* [37] as the deep learning framework and the weights obtained from the training procedure on the MS1M [33] and VGGFace2 [34] datasets. As reported in [13], we note that the use of a pre-trained model prevents overfitting phenomena that can occur due to the limited size of training datasets containing morphed images. For the backbone of the second block, we started from the SE-ResNet50 architecture trained on the VGGFace2 dataset. This dataset guarantees large variations in face age, illumination, pose, and ethnicity with more than 3 million faces belonging to more than 9 k different subjects. Then, this network was fine-tuned for 30 epochs, using the *Stochastic Gradient Descent* (SGD) optimizer, with a learning rate equal to 10^−4^ and a momentum of 0.9. No early stopping techniques were applied.

In the second step, the whole framework was trained in an end-to-end manner, freezing the weights that belonged to the backbones of each block, helping to avoid overfitting on them. Specifically, this training step was divided into two sub-phases. In the first one, only the Identity block was trained, i.e., the Artifact block and the following MLP were frozen. Then, in the second phase, also the Artifact block and the following multilayer perceptron were included in the training. This particular training procedure (different, for instance, from training all networks at the same time), led the framework to avoid unwanted side effects, such as the neglect of one of the block’s features from the final classification. In both training procedures, we conducted training operations using the *Adam* [38] optimizer, with an initial learning rate of 10^−3^ and a weight decay of 10^−4^. In our experiments, we trained the Identity block for 10 epochs and the Artifact block, in the second step, for 2 epochs.

## 4. Results

In this section, the experimental results of the proposed framework are reported and discussed. In particular, we firstly analyzed the performance in the D-MAD scenario, that is the scenario directly addressed by the proposed framework. Following, we report an investigation about the performance in the S-MAD scenario, using a single backbone of the Artifact block in order to predict if a single face image is morphed or bona fide.

### 4.1. Datasets

For our experimental evaluation, we used three different datasets: the *Progressive Morphing Database* (PMDB) [11], the *MorphDB* [11], and the *AMSL Face Morph Image* [39] datasets. We conducted a cross-dataset evaluation, exploiting the PMDB dataset only for the training phase, while performance was computed on the *MorphDB* and the *AMSL Face Morph Image* datasets.

#### 4.1.1. Progressive Morphing Database

The PMDB dataset consists of more than 1000 facial morphed images obtained through the morphing algorithm described in [11]. The original facial images were collected from the well-known AR [40], FRGC [41], and Color Feret [42] databases and belong to 280 subjects (134 males and 146 females). Morphed images are automatically created: this element guarantees a large number of samples available. Nevertheless, the absence of any manual retouch limits the overall quality of morphed images, and visible artifacts, such as texture inconsistencies, ghosts, and blurred areas, are present and visible, especially in the area of the eyes, mouth, and face contour (see Figure 1). Further details of the dataset were reported in [11]. We used this dataset in the training procedure, splitting data into training and validation subsets in a cross-subject manner. Specifically, we put 225 subjects (108 males and 117 females) in the training set and the remaining 55 (26 males and 29 females) in the validation set. In this way, during the training procedure, the network was forced to learn features related to the presence of artifacts and not related to the identity of the subjects.

#### 4.1.2. MorphDB

**Figure 3:**
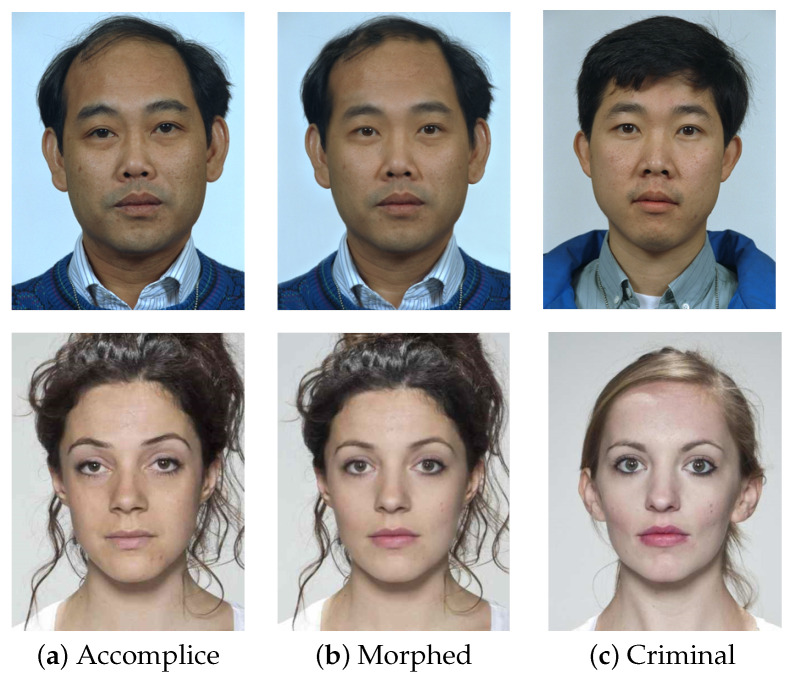
Sample images from the MorphDB (first row) and AMSL (second row) datasets. The final morphed image, along with the accomplice and the criminal starting images, is reported, in order to appreciate the different morphing factor used for the dataset generation. It can be noticed that the morphed image in AMSL is a balanced mix of both original subjects, while it more similar to the first subject in MorphDB.

The MorphDB dataset was created starting from the images collected in the FRGC [41] and Color Feret [42] datasets. It consists of 100 morphed images obtained from 100 different subjects (50 males and 50 females). For each morphed image, the two original images and a variable number of test images are also included. The *Sqirlz Morph 2.1* [43] algorithm was used to generate morphed images with a morphing factor in the range [0.3, 0.4]. These images were then manually selected in order to maximize the probability of fooling FRSs and human experts (such as the officer at the passport issuing stage). Moreover, morphed images were manually retouched to remove at best any visible artifacts. In this way, MorphDB is different from the PMDB dataset, since it contains high-quality morphed images without evident artifacts. This element led us to use this dataset during the testing phase. Further details of the dataset were reported in the original work [11], while some sample images are reported in the first row of Figure 3.

#### 4.1.3. AMSL Face Morph Image Data Set

This dataset [39] consists of morphed images generated from two different sources. The first one is the *Face Research Lab London Set* [44], while the second one is the *Utrecht ECVP Dataset* [45]. All face images comply with the requirements of the ICAO portrait quality [46]. A total number of 2175 morphed images were generated through the approach reported in [47], with a morphing factor equal to 0.5, belonging to 102 adult faces. All the generated images were created to fit on a single chip of the eMRTD [48], then they were compressed with the JPEG2000 algorithm, and the maximum size of each image was 15 kB. We note that this dataset is different from the previous ones, since JPEG2000 compression tends to delete most of the visible artifacts introduced during the morphing procedure. The second row of Figure 3 contains some images from this dataset.

### 4.2. Metrics

The experimental results are reported exploiting the *Bona Fide Presentation Classification Error Rate* (BPCER) and *Attack Presentation Classification Error Rate* (APCER) metrics, commonly used for the MAD scenarios. Given *N* bona fide images, the BPCER is computed as the percentage of images falsely classified as morphing presentation attacks. Similarly, given *M* morphed images, the APCER is computed as the percentage of images falsely classified as bona fide. From a mathematical point of view, these metrics are expressed through the following equations:

(1) $$\mathrm{BPCER}\left(\tau \right)=\frac{1}{N}\sum_{i=1}^{N}H\left(b_{i}-\tau \right)$$

(2) $$\mathrm{APCER}\left(\tau \right)=1-\frac{1}{M}\sum_{i=1}^{M}H\left(m_{i}-\tau \right)$$

where $b_{i}$ and $m_{i}$ are detection scores on bona fide and morphed images, respectively, τ is a score threshold, and H(x) is the unit step function defined as: H(x)={1ifx≤0, 0otherwise}. In addition, we expressed the BPCER also with respect to a defined value of APCER: BPCER_100_ and BPCER_1000_ represent the lowest BPCER related to APCER ≤ 10% and APCER ≤ 1%, respectively. We report also the *Equal Error Rate* (EER), the point at which BPCER and APCER assume the same value, and the *Detection Error Trade-off* (DET) curve, in which APCER and BPCER are plotted.

### 4.3. Experimental Evaluation in the D-MAD Scenario

The proposed framework was tested on the MorphDB and AMSL datasets in the D-MAD scenario, i.e., two facial images were given as the input. In addition, we compared our system with recent approaches available in the literature. As a first competitor, the approach presented in [17] was implemented; it investigates the use of several hand-crafted features in combination with an SVM classifier. In our implementation of this work, we initially tested all features and selected LBP features since this achieved the best performance on the test sets. For the second competitor, the method [21] proposed for the S-MAD scenario was adapted to work in the case of the differential scenario: it was based on a combination of features computed on a single image that was used to train an SVM classifier. To adapt this work, we computed descriptors for both input images, and we combined them to train and test an SVM classifier. Moreover, we implemented the current state-of-the-art method proposed by Scherhag et al. in [13], based on an SVM trained to classify the features computed by the ResNet50 architecture described in [14], extracted from two input images. Finally, for the AMSL dataset, we report the results of the method described in [16] taken from the original paper.

Results are reported in Table 1 and Table 2. All methods were tested on two types of couples, as indicated in the first column of both tables: “criminal” means that the probe image was associated with the image that belonged to the criminal, while “accomplice” means that the trusted image belonged to the accomplice. “Both” denotes that all the aforementioned couple types were merged into a single set. The morphing process, which merges two faces into a single one, was based on the morphing factor (α) that weights the presence of the two original subjects. Therefore, if α is different from 0.5, the final morphed image will be more similar to one of the two subjects. The choice of the correct α is a key element for the success of the morphing attack. In order to fool the human officer during the passport issuance, the morphed photo must be similar enough to the accomplice (the person requesting the document). On the contrary, during the control at automated gates, the morphed photo must be similar to the criminal [49] (the person that carries the attack thanks to the accomplice’s collaboration). Therefore, the use of different types of couples (i.e., different values of α) is interesting and useful to monitor the performance of MAD systems during all the steps involved and required in face morphing attacks.

**Figure 4:**
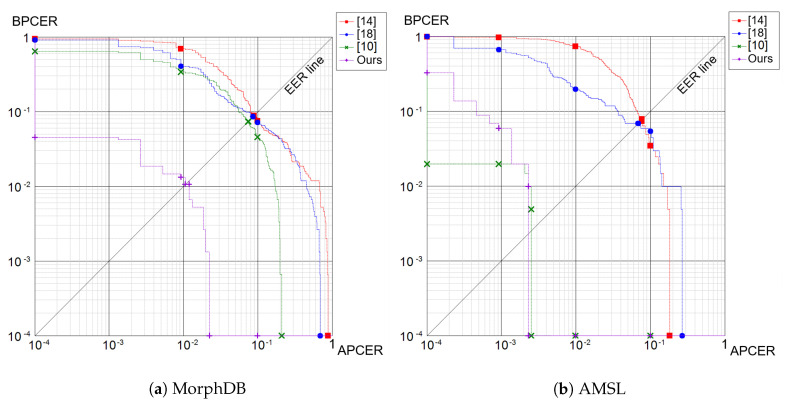
Detection Error Trade-off (DET) curves computed on the MorphDB (**a**) and AMSL (**b**) datasets.

As reported in Table 1, the method described in [13] and the proposed framework obtained high accuracy in the “criminal” scenario. Probably, in this setting, features related to the identity were sufficiently discriminative, and then, the classification was effective. Different results were instead obtained in the “accomplice” scenario, in which the classification based only on identity features was not so reliable: we observed that [13] had a dramatic change in performance. In contrast, the proposed framework seemed to be able to combine identity-based and artifact-related features and overcame the competitors in all the metrics reported. Similar observations can be made also for the AMSL dataset (Table 2), in which the performance in the “criminal” setting of our method and that of [13] were close. The “accomplice” scenario presented similar results, in terms of EER, to the “criminal” one: while in the MorphDB dataset, morphed images were very similar, in terms of identity, to the accomplice, in the AMSL dataset, morphed ones were halfway between criminal and accomplice, as depicted in Figure 3. Overall, this was due to the different morphing factors adopted during the creation of these two datasets, which influenced the proportion of the criminal and accomplice in the final morphed face. Finally, we note that the introduction of JPEG2000 compression negatively affected the performance of the proposed framework, since artifacts tended to be deleted. A general overview of the obtained results is depicted with the *Detection Error Trade-off* (DET) curves reported in Figure 4.

### 4.4. Ablation Analysis

As detailed before, the proposed framework was based on two different modules, i.e., the Identity and the Artifact blocks, respectively. Here, we conducted an ablation study, testing the performance of each single module to investigate its contribution towards the final morph detection score. Specifically, we removed the last fully connected layer (see the right part of Figure 2), used to merge features computed by both modules, using only the MLP to predict the final score. Experimental results are reported in Table 3 and were computed on couples created with both criminal and accomplice images. As shown, separated modules generally performed worse than the whole framework, since they relied on a backbone trained to extract a specific type of feature. Moreover, we observed that the MorphDB dataset was more challenging in terms of face verification complexity (the EER of the Identity block was higher), while the AMSL dataset was more challenging in terms of artifact detection, due to the JPEG2000 compression applied to its images. Therefore, the last fully connected layer of the framework was a key element in order to merge the information of each module, allowing balancing the single contributions and achieving high performance on both datasets.

### 4.5. Analysis of the Siamese Backbone

The choice of the backbone to be used in the blocks was a key element. Indeed, the backbones of the Siamese networks played a crucial role, since they extracted the features from the single images, and these features were then processed by the following components of the proposed framework. Then, in this section, we focused on the backbone adopted for the Artifact block, since the implementation and the training procedure of the Identity block’s backbone followed the guidelines of the original paper [14] and of the method proposed by Scherhag et al. [13].

**Figure 5:**
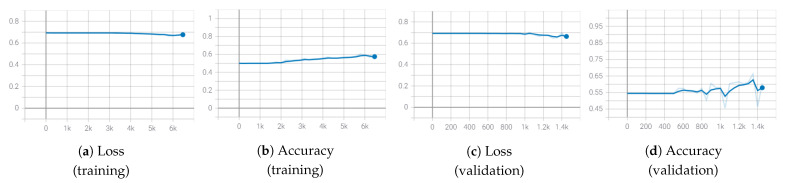
Train and validation loss and accuracy of the SqueezeNet network trained on the PMDB dataset. In blue, values with the training from scratch, while in gray, values for the training started from the pre-trained network.

Firstly, we analyzed whether, given the PMDB dataset as the training set, it was possible to train a deep neural network from scratch in the S-MAD scenario. This experimental evaluation was conducted adopting a shallow and limited-in-depth neural network, i.e., SqueezeNet [50], that was able to achieve good classification performance with fewer parameters and a limited model size with the respect to many deep architectures available in the literature. In Figure 5, we report the graphs of the accuracy and the loss values on both the training and validation sets obtained by training the network from scratch (i.e., with initial random weights). As shown (in blue), the limited size and image variety of PMDB dataset strongly limited the growth of the accuracy percentage and the related decrease of the loss values. On the contrary, the same SqueezeNet architecture pre-trained on the ImageNet dataset [51] was able to efficiently learn, as shown by the values reported in gray. Therefore, for all the experiments reported in this paper, we used pre-trained deep neural networks fine-tuned on the PMDB dataset.

A second step of the analysis was to determine the architecture that could provide the best results for our task. The second column of Table 4 reports a list of network candidates with the related pre-trained weights available in the literature (and, specifically, for the *PyTorch* deep learning framework). For all these architecture, we performed a fine-tuning stage on the PMDB dataset, and we tested them on the MorphDB dataset. There was a group of networks trained on the well-known ImageNet dataset and a group trained on the data provided by two datasets, i.e., VGGFace2 and MS1M. As expected, given a dataset, the final accuracy varied relying on the type of architecture exploited. Moreover, we note that the training on the VGGFace2 and MS1M datasets achieved the best accuracy. Therefore, we adopted the SE-ResNet50 architecture pre-trained on these two datasets as the backbone for the Artifact block of our framework.

**Figure 6:**
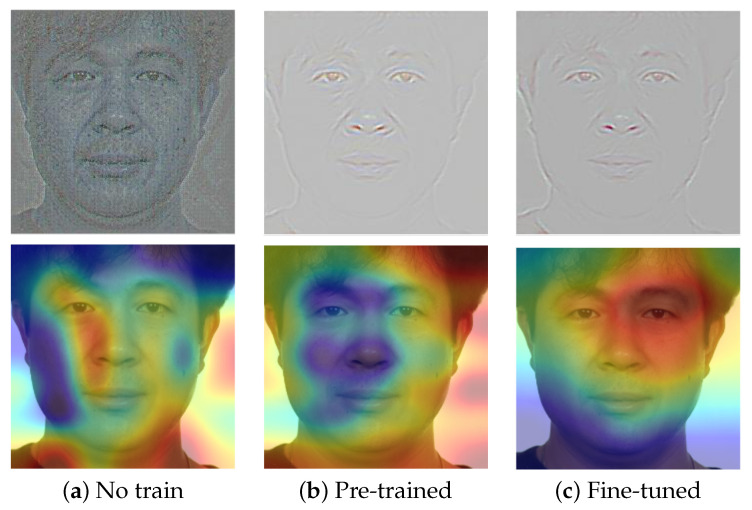
Visual outputs of the *guided backpropagation* (first row) and *Grad-CAM* (second row) algorithms that show the activation of the *SqueezeNet* architecture used for the S-MAD analysis (see Section 4.5).

Finally, we investigated the behavior of the networks exploiting the *Grad-CAM* [53] and the *guided backpropagation* [53] algorithms, as implemented in [54]. These algorithms were proposed in order to visualize and explain the internal behavior of neural networks or, rather, to visualize the gradient activations given an input image of a specific class. Some examples are reported in Figure 6, in which in the first row, we report the visualization of the guided backpropagation (in which colors and contours reveal a high activation of the gradient), while in the second row, the images produced by the Grad-CAM algorithm (in which colors tending to red denote high activation values) are reported. In the first column, we show the result obtained with the SE-ResNet50 architecture randomly initialized and not trained. As depicted, the network was not able to focus on specific facial areas, but on the contrary, it took into account arbitrary locations, such as the background and the neck. In the second and third columns, visual results with the pre-training and fine-tuning procedures, respectively, are shown and indicate that the network focused on a specific face element, and in particular, with the fine-tuning procedure, it concentrated attention on salient central face regions. While we observed a similar behavior on a number of different images, more studies will be necessary to interpret the model decisions.

### 4.6. Preliminary Results on P&S Images and GAN-Generated Morphed Images

In this paper, we focused on digital images, i.e., the training, validation, and test procedures were conducted on the digital images available in the publicly released datasets. We performed some preliminary tests also on printed and scanned (P&S) images, i.e., images that were acquired, printed, and finally scanned: this is the typical scenario in which the ID photo is provided by the citizens printed on photographic paper and then scanned by the officer during the issuing procedure. As mentioned above, this process introduces significant changes in images and then strongly influences the accuracy of MAD systems. To perform this test, we exploited the P&S images present in the MorphDB dataset (unfortunately, P&S images are not available in the AMSL dataset), obtaining an EER of 7.14% (BPCER_100_ = 23.4%, BPCER_1000_ = 38.7%) on MorphDB. The accuracy of the framework significantly decreased, even though we note that our framework was not directly trained or fine-tuned on P&S images.

We also investigated the performance of the proposed framework with images generated through a GAN-based approach. Specifically, we used the method described in [55] starting from the criminal and accomplice images available in the MorphDB dataset. The proposed framework achieved an EER of 9.33% (38% BPCER_100_ and 54% BPCER_1000_), despite it not being trained on datasets generated through GAN approaches (i.e., the network never learned to detect artifacts related to GAN-based generation procedures).

These preliminary tests led us to consider the training data. As described before, the Artifact block, and specifically its backbone, learns to detect artifacts similar to those present in the training set. As a consequence, during the testing phase, the network will correctly detect artifacts already seen during training. Therefore, the choice of the training dataset is a key element in order to detect certain types of artifacts. Since the underlying idea of the proposed method was to investigate the combined use of features related to the identity and artifacts, the choice of training datasets should be made taking into consideration the variety of morphed images and the use of different morphing tools.

## 5. Conclusions and Future Work

The proposed framework tackled the D-MAD scenario obtaining satisfying performance on both the MorphDB and AMSL datasets. However, we note that the training phase and the input data played a crucial role in the final performance. In particular, the system was able to detect artifacts to the extent that they were present in the training set. In addition, compression and other procedures, such as the P&S operation, could severely affect the quality of input images and then the performance of the framework. These limitations can be addressed through the acquisition of new datasets, created with a variety of face morphing tools, and the adoption of particular data augmentation techniques, which will be investigated in future work. We believe that this work could represent a first step towards the definition of a D-MAD strategy that aims to combine identity- and artifact-related features in a fully deep learning-based pipeline. The proposed framework was tested following a cross-dataset evaluation, improving performance especially in the presence of the couple composed of the morphed face and the accomplice. Plenty of future works can be planned, ranging from the deeper investigation about the use of P&S- and GAN-generated images in the training and testing sets, to the implementation and comparison with recent methods proposed in the literature. Finally, we will prepare a submission for the FVC OnGoing [56] and NIST platforms. Unfortunately, this requires a specific software implementation that is not easy to fulfill and to meet the strict time constraints.

## Figures and Tables

**Table 1:** Experimental results obtained on the MorphDB [11] dataset. In the first column, we report the type of couple that is created with the probe image and the image of the criminal or the accomplice (or both), leading to different scenarios. For further details about the metrics reported, see Section 4.2.

Couple Type	Method	EER	BPCER_100_	BPCER_1000_
Criminal	[17]	6.78%	66.4%	92.9%
	[21]	6.01%	37.8%	72.2%
	[13]	**0.00**%	**0.00**%	**0.00**%
	Ours	**0.00**%	**0.00**%	**0.00**%
Accomplice	[17]	11.1%	80.7%	91.9%
	[21]	10.5%	51.2%	90.7%
	[13]	11.5%	49.7%	65.5%
	Ours	**1.42**%	**1.85**%	**4.50**%
Both	[17]	8.83%	69.3%	93.8%
	[21]	8.57%	40.4%	90.8%
	[13]	7.37%	33.8%	63.9%
	Ours	**1.06**%	**1.32**%	**4.50**%

**Table 2:** Experimental results obtained on the AMSL [39] dataset. In the first column, we report the type of couple that is created with the probe image and the image of the criminal or the accomplice (or both), leading to different scenarios. The results of [16] ${}^{a}$ were obtained when the trusted image was known to the detection framework.

Couple Type	Method	EER	BPCER_100_	BPCER_1000_
Criminal	[17]	8.92%	85.3%	98.0%
	[21]	5.86%	14.7%	66.7%
	[13]	**0.00**%	**0.00**%	**0.00**%
	Ours	0.14%	**0.00**%	5.88%
Accomplice	[17]	5.93%	56.9%	83.3%
	[21]	6.86%	25.5%	59.8%
	[13]	0.25%	**0.00**%	**1.96**%
	Ours	**0.09**%	**0.00**%	**1.96**%
Both	[17]	7.50%	75.5%	96.6%
	[21]	6.86%	19.6%	66.2%
	[16]	3.11%	-	-
	[16] ${}^{a}$	2.36%	-	-
	[13]	0.13%	**0.00**%	**1.96**%
	Ours	**0.12**%	**0.00**%	5.88%

**Table 3:** Ablation study of the proposed framework. For each test dataset (MorphDB and AMSL), we individually tested the Identity and Artifact blocks, respectively, reporting the results obtained on couples with both the accomplice and criminal. For the sake of understanding, we show also the experimental results obtained through the whole framework as reported in Table 1 and Table 2.

Dataset	Module	EER	BPCER_100_	BPCER_1000_
MorphDB	Identity	8.20%	72.8%	99.4%
	Artifact	1.72%	19.0%	25.0%
	Both	1.06%	1.32%	4.50%
AMSL	Identity	1.10%	1.96%	5.88%
	Artifact	3.16%	6.86%	28.4%
	Both	0.12%	0.00%	5.88%

**Table 4:** Comparison of the performance in the S-MAD scenario with respect to a variety of architectures and datasets for the pre-training phase. All networks were then fine-tuned on the PMDB dataset and tested on MorphDB.

Dataset	Network	EER	BPCER_100_	BPCER_1000_
VGGFace2 [34]	Se-ResNet50 [35]	**1.75**%	**1.50**%	**7.00**%
MS1M [33]	ResNet50 [32]	2.75%	5.00%	13.50%
ImageNet [51]	AlexNet [25]	6.50%	100.00%	100.00%
	ResNet18 [32]	5.25%	17.50%	25.00%
	VGG-19 [24]	**2.75**%	**9.50**%	27.50%
	MobileNet [52]	6.00%	24.50%	35.00%
	SqueezeNet [50]	6.25%	11.00%	**13.00**%

## Data Availability

AMSL dataset is available at https://omen.cs.uni-magdeburg.de/disclaimer/index.php (accessed on 13 May 2021).
